# Association between epidural-related maternal fever and short-and long-term prognosis of parturients: A prospective observational study

**DOI:** 10.3389/fsurg.2022.1064272

**Published:** 2023-01-10

**Authors:** Bing Li, Yicong Liao, Qingning Wang, Shiyuan He, Lulu Yang, Junxiang Jia, Baisong Zhao

**Affiliations:** ^1^ Department of Anesthesiology, Women and Children’s Hospital, School of Medicine, Xiamen University, Xiamen, China; ^2^ Department of Anesthesiology, Guangzhou Women and Children’s Medical Center, Guangzhou Medical University, Guangzhou, China

**Keywords:** epidural-related, maternal, fever, labor epidural analgesia, long-term

## Abstract

We aimed to explore the association between epidural-related maternal fever (ERMF) and prognosis of parturients. 159 parturients who underwent vaginal delivery under labor epidural analgesia (LEA) received noninvasive continuous core body temperature monitoring. 122 of them completed the 42-day postpartum follow-up. Parturients with body temperature ≥38°C during labor were categorized as the Fever group, while the others were categorized as the No-Fever group. Compared to No-Fever group, Fever group had a greater proportion of primiparas, greater gestational age of parturients, and longer third stage of labor. The cesarean section and forceps delivery rates, and the amount of intrapartum hemorrhage in Fever group were significantly higher. There were no significant between-group differences with respect to puerperal infection, and amniotic fluid turbidity degree, neither significant between-group difference at 42-days postpartum. We found that ERMF was associated with some short-term outcomes. However, it showed no relation with long-term prognosis of the parturients at 42-days postpartum.

## Introduction

Intrapartum maternal fever, defined as oral temperature ≥38°C during childbirth (1), is known to be associated with increased incidence of postpartum adverse outcomes (2), such as prolonged labor, increased rate of instrumental delivery, early postpartum hemorrhage, and increased probability of placental retention (3). Labor epidural analgesia (LEA) is one of the most common non-infective cause of intrapartum maternal fever (4), and approximately 20% of pregnant women who receive LEA would have epidural-related maternal fever (ERMF) (5). LEA duration is corelated with ERMF; LEA greater than 6 h was shown to increase the risk of fever during labor (6). Whether ERMF needs intervention or not depends on the harm it causes to the mother and newborn. Therefore, a comprehensive understanding of the harmful effects of ERMF is of much clinical significance.

ERMF may further affect the prognosis of the parturient in the postpartum period by changing the delivery experience of the parturient and the outcomes of the newborn. However, whether ERMF affects the short-term and long-term prognosis of parturients is seldom reported. In this study, we investigated the short-term and long-term (42 days postpartum) outcomes of parturients who received LEA at a single center. The objective was to explore whether ERMF is associated with the short-term or long-term prognosis of parturients.

## Materials and methods

### Research population

This study was approved by the Ethics Committee of the Guangzhou Women and Children's Medical Center (Protocol number: 201939000) and registered at the Chinese Clinical Trial Registry (http://www.chictr.org.cn/edit.aspx; ID: ChiCTR1900025653; Principal investigator: Baisong Zhao; Date of registration: Sep 5, 2019). This study adhered to the STROBE reporting guidelines. This study recruited pregnant women who planned to give birth during daytime hours from 30 September 2019 to 31 December 2019 and came to our hospital for treatment. All participants provided written informed consent before the start of the trial.

The inclusion criteria were as follows: (1) parturients without fever before labor; (2) parturients with full-term singleton pregnancy (gestational age >37 weeks); (3) vaginal in-hospital delivery; (4) vertex presentation; (5) no risk of mother-to-child transmission of an infectious disease; (6) no suspected fetal coagulopathy; (7) parturients without prenatal vaginitis; and (8) willingness to use LEA and ability to provide informed consent. The exclusion criteria were: (1) both upper extremities required for venous access; (2) absence of both upper limbs; (3) history of severe heart disease like myocardial infarction, heart failure; (4) history of severe infectious diseases such as maternal hepatitis, or HIV infection; (5) history of serious skin allergies, especially allergy to silicone or plastic; (6) preeclampsia; (7) parturients who took paracetamol within 6 h before receiving epidural analgesia; (8) suspected fetal coagulopathy; and (9) chorioamnionitis confirmed by pathological examination.

Upon entering the delivery room, the researchers provided all eligible women with information about the study and obtained their informed consent for participation in the study. All parturients were monitored continuously. Patients with body temperature ≥38°C during labor were categorized as the Fever group while those with body temperature <38°C were categorized as No-Fever group. The data collection process was blinded, and researchers who collected body temperature data had no knowledge of the duration and dose of LEA. In addition, personnel involved in acquisition of postpartum data were blinded to maternal body temperature.

### Measurement method of body temperature and fever treatment

A wireless thermometer, iThermomonitor (Rui Ren Medical, China), connected to the central control platform was used to carry out axillary temperature measurement during the whole delivery process (10 values per second). The iThermonitor was fixed to the shaved armpit using an adhesive tape with low risk of allergic reaction. Women were instructed to maintain the arm in the adducted position for up to 5 min until the temperature value shown on the display was stable; thereafter, the women were allowed free movement of their arms (7). Generally, intravenous infusion of unwarmed fluid into the contralateral arm does not have any effect on the use of iThermonitor for measuring the axillary temperature.

The pregnant women reported the onset of delivery. Labor room nurses were responsible for monitoring the patients and executing the obstetrician's orders, who did not participate in the study. Fever was defined as axillary temperature (measured by iThermonitor) ≥38°C. In parturients with fever, the following treatment measures were undertaken: When axillary temperatures rose to 38°C–38.5°C, intravenous rehydration was accelerated to replace water loss. When the axillary temperature exceeded 38.5°C, Tylenol suspension was administered orally (Johnson & Johnson Pharmaceutical Ltd., China).

### Implementation of epidural analgesia

Epidural analgesia was initiated once uterine contractions were regular and cervical dilatation was ≥1 cm, and was continued to 2 h after the end of labor. Women receiving LEA were administered intravenous Ringer's lactate infusion (2–4 ml/kg/h). The rescue drugs atropine and ephedrine were always kept prepared. An epidural catheter was placed at the L3–L4/L2–3 and epidural analgesia was controlled by the parturients themselves. After the epidural catheter was fixed, a test dose of 4 ml of 1% lidocaine was administered *via* the catheter. Five minutes later, in the absence of any side effects, a 10 ml bolus dose of ropivacaine 0.0625% (lot number: H20140763; Astra Zeneca, Sweden) plus 0.3 µg/ml sufentanil (batch number: H20054256; Yichang Renfu Pharmaceutical, China) was administered. Subsequently, the epidural catheter was connected to the pulse pump and set as follows: loading dose, 10 ml; injection rate, 6 ml/h; PCA dose, 8 ml; maximum dose, 40 ml/h; and lockout interval, 10 min. At any time during labor, women with excessive pains or a visual analogue scale pain score >3 were administered an additional 5 ml of ropivacaine 0.1%.

### Data collection

Data for the following demographic and basic clinical characteristics were collected from all enrolled women: gestational age, height, weight, body mass index (BMI), and time of delivery. Delivery-related data included the duration of labor, neonatal Apgar scores, delivery outcomes, amount of postpartum hemorrhage, occurrence of puerperal infection, amniotic fluid turbidity, lochia, and menstruation at 42 days after delivery. We excluded data of patients with pathologically confirmed chorioamnionitis.

Maternal blood pressure and heart rate was continuously monitored during labor and the rate and volume of fluid administered was administered.

### Statistical analysis

The required sample size was calculated based on the ratio of abnormal delivery in parturients who did not develop fever during labor and those who developed fever in our previous trials. According to the ratio of 1:3 and with *α *= 0.05 and power of 0.9, the minimum sample size required for the No-Fever group was 111 and that for the Fever group was 37.

Use SPSS software (ver. 21.0; SPSS Inc., USA) was used to conduct statistical analysis. Owing to the non-normal distribution of gestational age, height, weight, BMI, amount of hemorrhage during labor and 2 h after delivery, these data are expressed as median and interquartile range [M (Q)] and the between-group differences assessed using the rank sum test. Categorical variables are expressed as frequency and percentage [*n*(%)] and between-group differences assessed using the Chi-squared test. *P* values < 0.05 were considered indicative of statistical significance. The duration of labor and Apgar score are expressed as mean ± standard deviation and between-group differences assessed using repeated measures analysis of variance. When the differences were statistically significant, the least significant difference (LSD) method was further used to make pair-wise comparisons.

## Results

**Figure 1:**
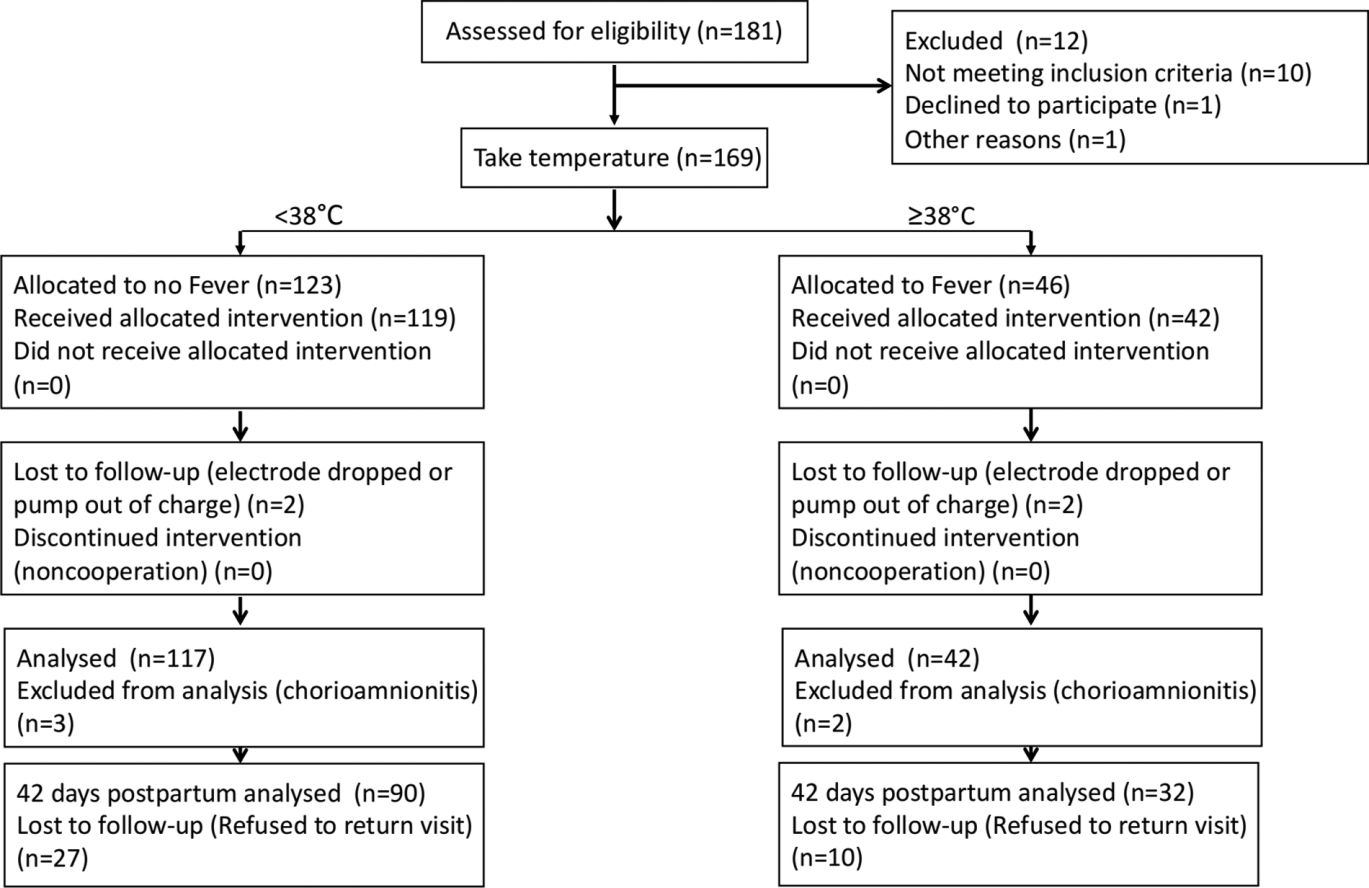
Experimental flow graph.

This study used a convenience sampling method and grouped parturients who received LEA into No-Fever group and Fever group according to axillary temperature. Finally, a total of 159 parturients who received LEA were recruited; of these 117 were in the No-Fever group (maximal temperature in the labor period <38°C) and 42 were in the Fever group (maximal temperature in the labor period ≥38°C). A total of 122 women completed the 42-day postpartum follow-up; of these, 90 were in the No-Fever group and 32 were in the fever group (Figure 1). For the maternal demographic and clinical characteristics, there were no significant between-group differences with respect to height weight and BMI. The women in Fever group were more likely to be primiparous, in addition, the gestational age of parturients in the Fever group was greater (Table 1).

**Table 1:** Maternal demographic and clinical characteristics.

Indicators	No-Fever (*n* = 117)	Fever (*n* = 42)	*x*^2^/*z*/*F*	*P*
Maternal type [*n* (%)]
Primipara	73 (62.4)	34 (81.0)	4.837	0.028
Multipara	44 (37.6)	8 (19.0)		
Gestational age [*M* (Q), weeks]	39.9 (1.6)	40.1 (1.6)	−2.171	0.030
Height [*M* (Q), cm]	160.0 (6.0)	160.0 (8.0)	−1.610	0.107
Weight [*M* (Q), kg]	66.0 (9.3)	68.0 (11.0)	−0.952	0.341
BMI [kg/m^2^, *M* (Q)]	25.9 (3.6)	27.1 (2.9)	−1.842	0.065

BMI, body mass index. *P *< 0.05 indicates significant between-group difference.

**Figure 2:**
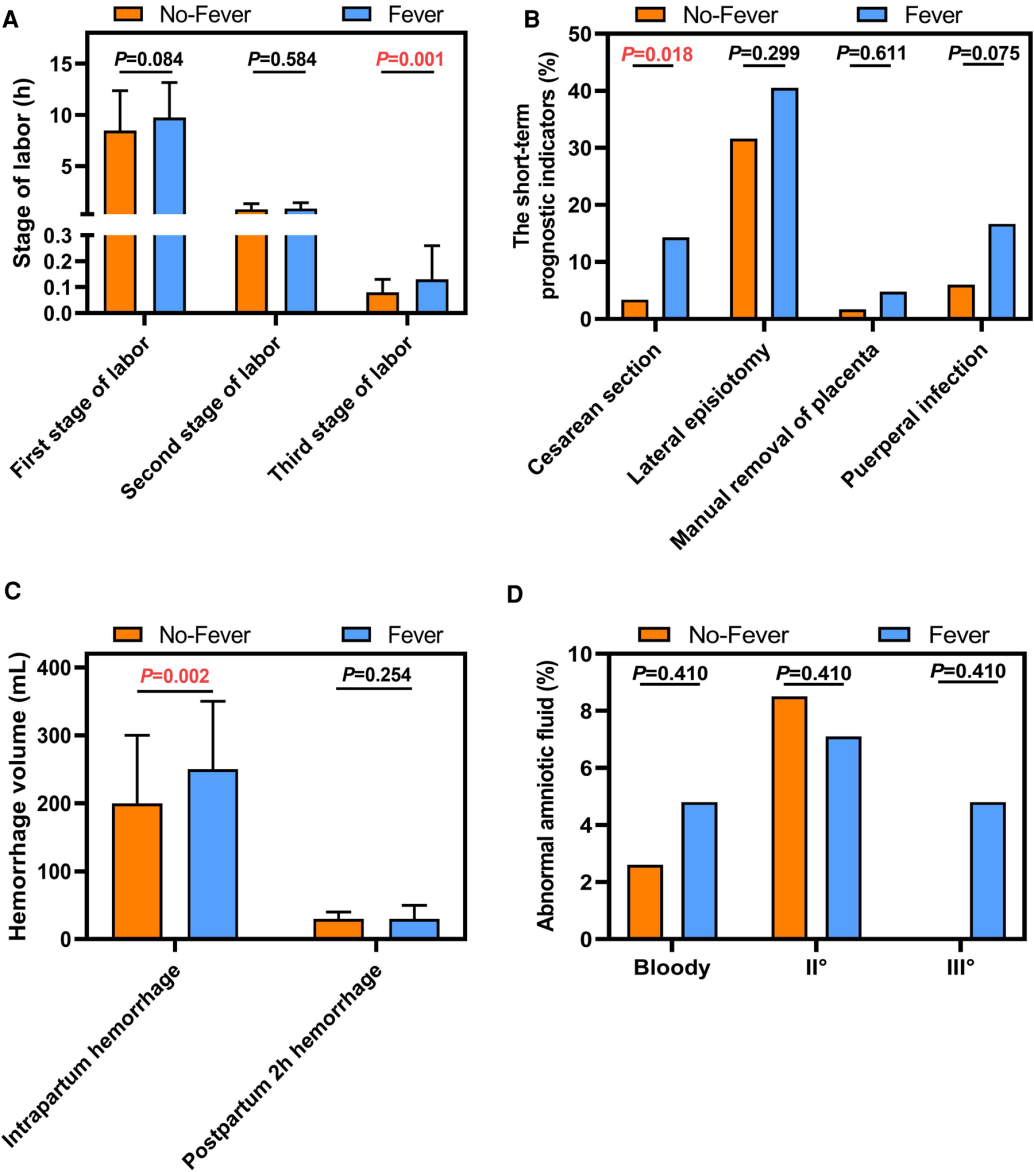
Comparison of short-term prognostic indicators between the two groups. (**A**) Stage of labor (h). (**B**) The short-term prognostic indicators (%). (**C**) Hemorrhage volume (mL). (**D**) Abnomal amniotic fluid (%).

There was no significant between-group difference with respect to the duration of the first (*P *= 0.084) and second (*P *= 0.0584) stages of labor. However, the duration of the third stage of labor in the No-Fever group was significantly shorter than that in the Fever group (*P *= 0.01) (Figure 2A). For the short-term prognostic, the cesarean section rate in Fever group was significantly greater than that in the No-Fever group (23.8% vs. 6.8%, *P *= 0.018). There were no significant between-group differences with respect to the rates of lateral episiotomy, manual removal of placenta, and puerperal infection (Figure 2B). The amount of intrapartum hemorrhage in the Fever group was also significantly greater than that in the No-Fever group (*P *= 0.002); however, there were no significant between-group differences with respect to the amount of hemorrhage in 2 h postpartum (Figure 2C). Moreover, there were no significant between-group differences with respect to the degree of turbidity of the amniotic fluid (Figure 2D). These results suggest that ERMF affects the short-term prognosis of the puerpera.

**Figure 3:**
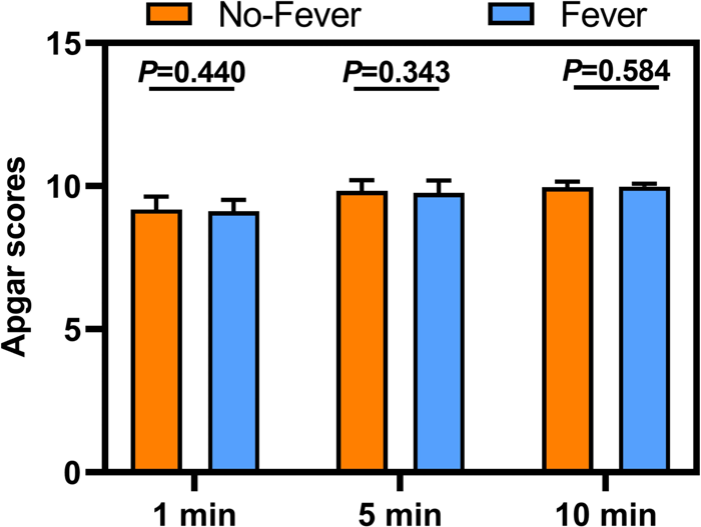
Apgar scores between the two groups.

**Figure 4:**
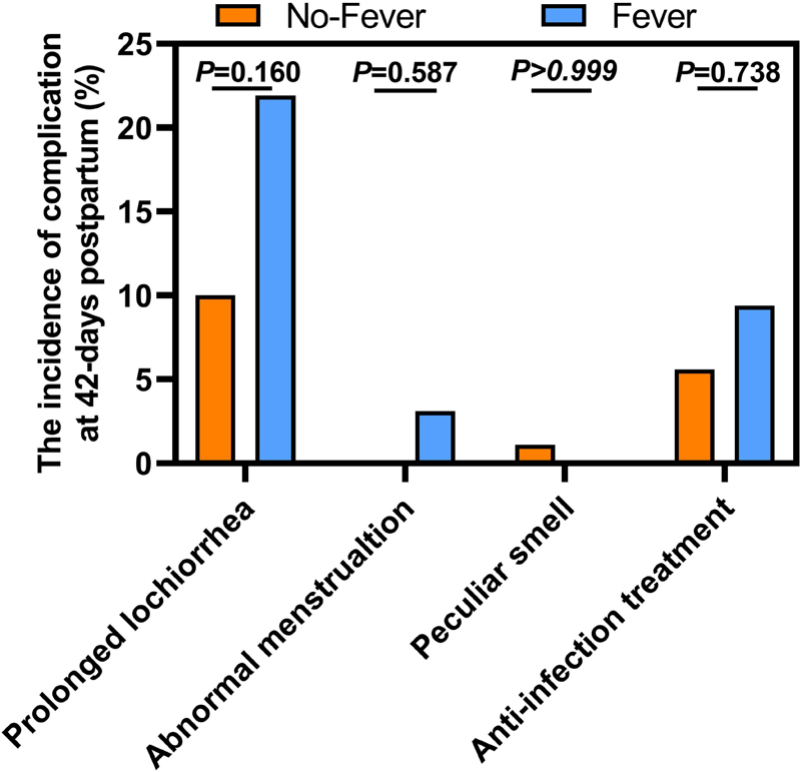
Comparison of outcomes at 42-days postpartum between the two groups.

From 1 to 10 min, there was no significant difference in the Apgar scores between the two groups (Figure 3). For the long-term prognostic significance, there were no significant between-group differences with respect to lochia, menstruation, peculiar smell, and anti-infective treatment at 42-day postpartum follow-up (Figure 4). These results suggest that ERMF has no effect on the neonatal status and the long-term prognosis of puerpera.

## Discussion

This study observed the incidence of related short-term and long-term (42-day postpartum) adverse events in parturients who received LEA. The baseline characteristics were comparable in the two groups. The proportion of primipara and the gestational age (despite the difference of 2 days) in the Fever group were higher than those in the No-Fever group, while the third stage of labor in the Fever group was longer than that in the No-Fever group. The Fever group had a significantly higher cesarean section rate and rate of forceps delivery, while the rates of lateral resection and manual removal of placenta were comparable in the two groups. The amount of intrapartum bleeding was greater in the Fever group, while the amount of 2 h postpartum bleeding was comparable in the two groups. In addition, there was no significant difference in Apgar scores between the two groups. At 42-day postpartum follow-up, there was no significant between-group difference with respect to the proportion of women with clean lochia discharge, menstrual re-fluidity, secretions with unusual smell, and postpartum treatment with antibiotics.

In this study, we defined fever as core body temperature ≥38°C. Intrapartum fever is associated with increased incidence of postpartum adverse outcomes. In the study by Dior et al. (2), intrapartum fever significantly increased the probability of emergency cesarean section and device-assisted delivery, especially for parturients whose body temperature was >39°C. However, no causal association was observed on stratified analysis disaggregated by the degree of maternal fever. In the study by Ashwal et al. (3), fever during labor was an independent risk factor for maternal-related complications, and was aassociated with prolonged second stage of labor, increased instrumental delivery rate, cesarean section rate, and placental retention rate. The authors did not divide the women into groups according to whether they received LEA. In our study, we excluded parturients with infectious factors, and the rates of cesarean section and forceps delivery in the Fever group were higher than those in the No-Fever group; in addition, the Fever group had a greater amount of intrapartum hemorrhage. However, we also found no difference in the rate of puerperal infection and turbid amniotic fluid between the Fever and No-Fever groups. This indicated that the significant between-group differences with respect to the proportion of cesarean section and forceps deliveries, and the amount of intrapartum hemorrhage during delivery were not directly related to puerperal infection and might be related to ERMF.

Given the observational nature of study, we can only analyze the correlation of ERMF with increased rates of cesarean section and forceps deliveries and increased intrapartum bleeding. However, it is not clear whether ERMF directly affects the delivery outcomes, delays the delivery of placenta, increases the amount of intrapartum hemorrhage, or affects the delivery outcome. Of note, that there were also differences between the No-Fever and Fever groups with respect to the parity and gestational age of parturients. The proportion of primiparous women in the fever group was higher, and the gestational age was also greater (despite the difference of 2 days). Compared with primipara, the birth canal of the multiparas tends to be more relaxed, and the fetus also gains weight with increase in the gestational age. Studies have confirmed that primipara status (8) and larger neonatal birth weight (9) are risk factors for increased rates of cesarean section and forceps delivery. Excessive uterine dilation and uterine laxity caused by overweight fetuses (10), prolonged third stage of labor, and delayed placental delivery (11) are also risk factors for increased intrapartum bleeding.

At the same time, the impact of intrapartum fever on neonates has evoked increasing interest. The negative effects related to newborns mainly include some neurosuppressive symptoms, such as a decrease in the Apgar scores, the need for assisted ventilation and oxygen therapy, and even a rised chance of cardiopulmonary resuscitation (5, 12). Ashwal et al. (3), further divided the febrile mothers into blood and/or placental culture positive and negative groups. They found that the incidence of adverse neonatal outcomes in the positive group was much higher than that in the negative group, indicating that the negative impact of fever on newborns is more related to infectious factors. In this study, there was no difference in the puerperal infection rate between the Fever group and the No-Fever group; moreover, we observed no significant between-group difference with respect to amniotic fluid turbidity and the newborn Apgar score. This implies a lack of correlation between ERMF and neonatal hypoxia. Other studies conducted by our group (13) have also yielded similar results.

Uterine involution is usually completed within 42 days postpartum. Prolonged lochia or peculiar smell is a sign of poor postpartum uterine involution. In this study, parturients were followed up 42 days after delivery. We found no significant between-group difference with respect to these indicators. This suggests that ERMF may not affect uterine involution 42 days postpartum.

We still have some limitations in this study. This was a single-center, observational study with a relatively small sample size. A multicentre study with larger sample size and longer duration of follow-up is needed to provide more robust evidence.

In this study, ERMF was associated with prolongation of the third stage of labor, increased rates of cesarean section and forceps delivery, and increased intrapartum hemorrhage. However, ERMF was not found to affect the long-term prognosis at 42 days postpartum. This means that, different from infection-associated fever, ERMF has some association with short-term prognosis of parturients, but no impact on long-term postpartum recovery.

## Data Availability

The original contributions presented in the study are included in the article/Supplementary Material, further inquiries can be directed to the corresponding authors.
